# Safety concerns regarding viscoelastic delivery systems, or, caveat injector

**DOI:** 10.1038/s41433-025-03621-8

**Published:** 2025-01-24

**Authors:** Richard John Haynes, Panayiotis Maghsoudlou

**Affiliations:** 1 Department of Vitreoretinal Surgery, Bristol Eye Hospital, Bristol, UK; 2 Academic Unit of Ophthalmology, Translational Health Sciences, Bristol Medical School, University of Bristol, Bristol, UK

**Keywords:** Surgery, Business and industry

To the Editor,

Most ophthalmologists may assume proficiency in the usage of syringes. However, certain design elements in new devices, such as NuVisc Pro viscoelastic (BVI Medical, MA, USA), require strict adherence to handling instructions to avoid complications.

**Fig. 1:**
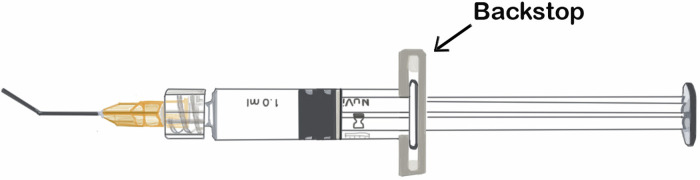
NuVisc Pro design indicating plastic backstop.

NuVisc Pro viscoelastic employs a glass syringe equipped with a plastic ‘backstop’—a component designed to support counter-pressure during injections (Fig. 1). This backstop is clipped onto a small flange on the glass syringe and can unexpectedly detach when pressure is applied e.g. when injecting viscoelastic into the eye.

The ‘Instructions for use’ leaflet specifies in paragraph 12, subsection ‘Instructions for the correct handling of the product’, point 6, in small print: “The syringe is held correctly if the backstop opens at the back, toward the hand that you are holding it in”.

Perhaps it is legally sufficient to have such a covert instruction to avoid potentially serious injury, but a perhaps it should also explain the reason, with something along the lines of: “failure to do so could result in sudden failure of the syringe resulting in it being propelled forward like an arrow from a crossbow”.

Such an adverse event occurred to one of our patients, wherein the plastic backstop detached during injection, resulting in posterior capsule rupture, iris root trauma and brisk intraocular bleeding. Dense vitreous haemorrhage persisted, requiring pars plana vitrectomy.

The manufacturer, BVI, is aware of the issue having previously acknowledged it through a Field Safety Notice issued on 12 September 2024 [1].

The notice states:

“Since May 2024, 4 complaints involving 5 incidents of NuVisc Pro, alleging the same medical device problem—‘backstop detached’ during surgery/injection or while preparing for surgery. Not following the Instruction for Use could lead to a backstop being detached from the syringe during injection, associated with the risk of a serious eye injury of the patient.”

In the section titled “Type of action to mitigate the risk,” the manufacturer states:

“Do not manipulate the backstop as this may cause instability in device handling during the procedure”

We have to question the logic of this instruction ‘not to manipulate the backstop’, because if there can only be one correct orientation of the backstop, how does a left-handed surgeon manage without ‘manipulating the backstop’?

In addition, the company states:

“Do not use if the backstop is loose or missing.”

This statement seems to be an admission that the backstop can be loose enough to fall off during transport.

This creates a paradox: How can anyone detect a loose backstop without touching it, when touching it violates the rule against manipulating backstops?

This incident raises critical questions about the adequacy of the device’s design and the communication of essential handling requirements. We suggest that BVI reconsiders the syringe design to enhance stability or, issues clearer, more accessible guidance for users to minimise risk.

Nota Bene: we have not carried out a survey of viscoelastic products from other manufacturers so it is possible this problem may not be exclusive to BVI’s NuVisc Pro. Caveat injector.
